# The Multifaceted Actions of CD73 During Development and Suppressive Actions of Regulatory T Cells

**DOI:** 10.3389/fimmu.2022.914799

**Published:** 2022-05-31

**Authors:** Meihong Da, Luxia Chen, Alexander Enk, Sabine Ring, Karsten Mahnke

**Affiliations:** Department of Dermatology, University Hospital Heidelberg, Heidelberg, Germany

**Keywords:** adenosine, regulatory T cells, immune suppression, CD73, immune checkpoint

## Abstract

Adenosine (Ado) has been shown to have immunosuppressive effects in a variety of diseases. It can either be released directly into the extracellular environment by cells, or it can be produced by degradation of ATP within the extracellular spaces. This extracellular pathway is facilitated by the concerted actions of the ectoenzymes CD39 and CD73. In a first step CD39 dephosphorylates ATP to ADP and AMP, respectively, and in a second step CD73 converts AMP to Ado. Thus, activity of CD73 on the cell surface of cells is the rate limiting step in the generation of extracellular Ado. Among T cells, CD73 is most abundantly expressed by regulatory T cells (Tregs) and is even upregulated after their activation. Functionally, the generation of Ado by CD73^+^ Tregs has been shown to play a role in immune suppression of dendritic cells, monocytes and T cells, and the defined expression of CD73 by Tregs in immunosuppressive environments, such as tumors, made CD73 a novel checkpoint inhibitor. Therefore, therapeutical intervention by anti-CD73 antibodies or by chemical inhibitors of the enzymatic function is currently under investigation in some preclinical animal models. In the following we summarize the expression pattern and the possible functions of CD73 in T cells and Tregs, and exemplify novel ways to manipulate CD73 functions in Tregs to stimulate anti-tumor immunity.

## Introduction

For long CD73 has been identified as a major adenosine (Ado) producing ectoenzyme expressed by different cell types. As Ado has profound immunosuppressive effects in a variety of diseases, there is a clear link between CD73 expressing cells and their immunosuppressive functions. In some cancers this relation even led to a correlation of the amount of CD73 expression with the severity of the disease. The defined expression of CD73 by tumor invading immune suppressive leukocytes and/or the tumor itself made CD73 a novel immune checkpoint molecule that may be targeted by specific inhibitors.

Among T cells, CD73 is most abundantly expressed by CD4^+^ CD25^+^ regulatory T cells (Tregs) and the generation of Ado by CD73^+^ Tregs has been shown to play a role in their immune suppressive action against dendritic cells, monocytes and effector T cells (1, 2). Therefore, therapeutical intervention by anti-CD73 antibodies (Abs) or by chemical inhibitors of the enzymatic function is currently under investigation in some preclinical animal models. In the following we summarize the expression pattern and the possible functions of CD73 *in vivo* and exemplify novel ways to manipulate CD73 functions in Tregs to stimulate anti-tumor immunity.

## The Structure of CD73

The ecto-5-nucleotidase, abbreviated as eN, e5NT or CD73 (gene NT5E, E.C. 3.1.3.5, UniProt P21589), is an enzyme that hydrolyses AMP. CD73 consists of a non-covalently linked homodimer and is attached to the outer cell membranes *via* glycosylphosphatidyl-inositol (GPI) anchors (3). The structure of human CD73 has been determined (4) and it revealed two distinct conformations (5). The binding of AMP occurs in an inactive (open) conformation, which enables the binding of the substrate to the C-terminal domain. The active (closed) conformation represents the active state where hydrolysis of the substrate occurs. This “closing” of the enzyme is accomplished by rotation of one domain (of nearly 100°), which brings the N- and C-terminal domains together forming the active site. By hydrolysis the phosphate group is cleaved from the ribose moiety and after switching back into the “open conformation” the product is released from CD73. Although this description is rather sketchy, it illustrates possible mechanisms of CD73 inhibition, as small molecules may be designed to interfere with the enzymatic activity by inhibiting the open–close process and/or binding to the defined region with higher affinity than the natural ligand AMP.

## The Basic Functions of CD73 During Steady State and Inflammation

But why may it be important to develop CD73 inhibitors? The reason for developing strong and specific CD73 antagonists derives from its Ado-producing properties. Ado, which constitutes one product of the enzymatic degradation of AMP by CD73, is a potent immunosuppressor (6). *In vivo*, CD73^+^ Tregs frequently co-express CD39, yet another ectoenzyme, which has comparable functions as CD73. CD39, however, fuels the CD73-derived production of Ado as it degrades ATP and ADP to AMP, which acts as substrate for CD73. This functional interdependence of CD39 and CD73 makes Tregs special as these cells express both molecules in sizable numbers (7) making them self-sufficient to create an Ado-rich, immunosuppressive pericellular environment, independent from other CD39^+^ cells (8).

**Figure 1:**
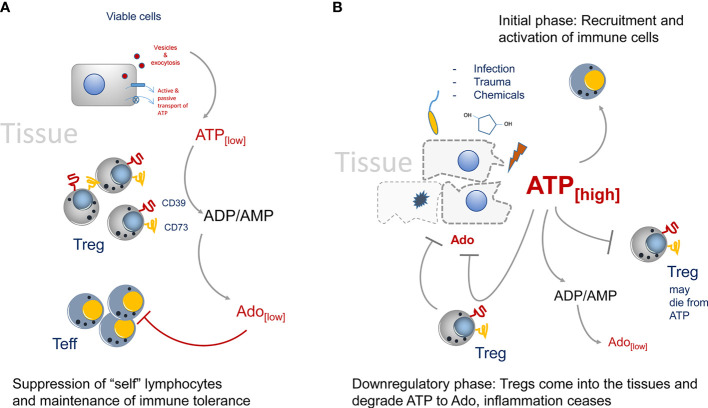
Regulation of CD73-mediated adenosine production in healthy and diseased tissues. **(A)** In the steady state only trace amounts of ATP are released by viable or by dying cells. It will be immediately degraded by CD73 to adenosine. Adenosine acts as immune suppressor and maintains immune tolerance by creating an immune suppressive environment. **(B)** The situation changes when infection, trauma and toxicity occur. Massive release of ATP cannot be degraded immediately by CD73. It activates lymphocytes and downregulates Treg populations, presumably by inducing cell death. Adenosine concentrations are kept low to maintain immunity. Later, more Tregs will enter the tissue side, degrading ATP and producing the immune suppressor adenosine.

In “steady state” situations, i.e. in absence of tissue disruption and infection, the presence of CD73^+^ Tregs in the environment helps to maintain the state of tolerance by producing Ado from extracellular ATP (**Figure 1A**). This immune suppression is rather weak, as only trace amounts of ATP are present in the tissue environment (9–11). In the absence of tissue damage, ATP can be released by cells *via* several mechanisms ranging from exocytosis to ATP-specific membrane transporters (12, 13). For instance, the vesicular nucleotide transporters VNUT or SLC17A9 can accumulate ATP in intracellular vesicles first, which can then subsequently be transported by exocytosis to the outside of cells (14). Such vesicle-mediated ATP release has been shown to be of importance during nutrient deprivation of cancer cells (15). Of interest, the ATP receptor P2x7 can stimulate the release of vesicles (16), offering the possibility that extracellular ATP promotes its own replenishment by this self-sustaining cycle.

Other non-exocytotic means of ATP release comprise ATP-binding cassette (ABC) transporters, eg. the multi drug resistance protein 1 (MDR-1), that hydrolyze ATP as energy source for the transmembrane movement of different molecules. These transporters do not only transport other molecules through membranes, they also can act as transporters for ATP (17). Finally, “classical” channels and pores in cells that allow ATP to cross the cell membrane are described. Among them are chloride ion channels (18), volume- and voltage-dependent anion channels (19), connexin hemichannels, pannexin 1, calcium homeostasis modulator 1, volume-regulated anion channels and maxi-anion channels (MACs) (20). Once outside of cells, ATP may act as a potent proinflammatory stimulus that engages P2x7 receptors, which can act as ATP channels themselves (21, 22), leading to activation of inflammasomes (23–25). Under homeostatic conditions extracellular ATP, although only present in trace amounts, needs to be readily degraded by CD39/CD73 to prevent the induction of an immune reaction. Additionally, the generated Ado may help even further to maintain a slightly immunosuppressive extracellular environment (26). Altogether this mechanism may be necessary to maintain a “healthy”, uninflamed tissue, as only few dying cells in the tissue must not induce an immune reaction (27).

However, the situation changes when trauma and/or infection in tissues occurs (**Figure 1B**). Now huge amounts of ATP are set free into the tissue environment and it will act as a potent immune activator (28). These relatively high amounts of ATP are necessary to trigger P2x7 ATP-receptors, which have an EC50 of approximately 1 mM. These concentrations can only be reached in diseased or necrotic tissues (29). Degradation of ATP to Ado lags behind and a potent immune reaction is generated. Only later in an inflammatory reaction Tregs will enter the tissue and due to their high expression of CD39 and CD73 they are strong producers of Ado, which will lead to downregulation of the inflammatory reaction. Of note, Tregs are highly sensitive to ATP concentrations. I.e., CD4^+^ CD25^+^ Tregs, as opposed to CD4^+^ effector T cells will die from high extracellular ATP levels (30). That way in the beginning of an inflammation immune reaction, the suppressive activity of Tregs may be switched off. That excess of extracellular ATP is indeed important to booster immunity can also be inferred from tumor bearing mice. It has been shown in tumor models that in P2x7 ATP-receptor deficient animals, more Tregs than effector T cells were recruited to the tumors, and that the Tregs were activated and highly suppressive as indicated by upregulation of the markers OX40, PD1 (31) and CD73. In summary, deletion of P2x7 signaling led to an immunosuppressive tissue environment (32, 33). However, at later stages of an inflammation, or after tumors have been largely eliminated, the extracellular ATP levels cease gradually by the activity of CD73^+^ and CD39^+^ cells. Among them are also double positive Tregs that will seed into the tissues and help to downregulate inflammation by ATP-to-Ado conversion, inducing the “healing” of the insult (34, 35).

## How Do CD73^+^ Tregs Suppress?

The main suppressive function of CD73^+^ Tregs relies on their capacity to produce Ado. However, CD73 expression does not make other suppressive means obsolete, as CD73^+^ Tregs still express their prototypic set of inhibitory receptors and Ado production just adds another means to an already existing arsenal of suppressive mechanisms (36, 37). Ado seems rather unstable and is thought to be degraded in tissues by adenosine deaminase within minutes (38). However, that was only investigated in dog’s serum and the exact distribution and half-life in tissues, other than blood, is not clear yet. Nevertheless, due to several Ado degrading mechanisms in tissues, Ado seems to be a short-range immune suppressor and therefore aligns with several other suppressive mechanisms of Tregs that require close cell-to-cell contact (i.e. CTLA-4, GITR) (36). At the same time, the short half-life of Ado restricts its suppressive effects to the local environment. Systemic immune suppression is therefore avoided.

Ado suppresses cells by engaging Ado receptors (AdoR) that can be divided into four distinct receptors, A1, A2_A_, A2_B_, A3 (39). They all belong to the G-protein-coupled-receptor (GPCR) family, but their intracellular signaling differs. A2-type AdoR are Gαs-protein coupled receptors, with the A2_B_ receptor additionally signaling *via* Gαq (40). After engaging AdoR, activated G protein complexes form at the inner cell membrane, leading to activation of the adenylate cyclase (AC) and to rising cAMP levels (in case of Gαs), finally activating protein kinase A (PKA) as secondary effector. On a molecular level this can directly be counteracted by engagement of A1 or A3 AdoR, which signal *via* Gαi/Gαq complexes, whereby the Gαi complex inhibits AC activity. However, the main pathway for signaling of A1 and A3 receptors is mediated by phospholipase C (PLC)-induced secondary messengers, eventually leading to a rise in intracellular Ca^2+^ and PKC activation. The common denominator of A2-type AdoR mediated suppression is cAMP as second messenger. Both A2-type AdoRs elevate cAMP levels by activating AC. Further downstream cAMP signals *via* PKA that regulates gene transcription *via* NF-κB, HIF-1α and CREB. In addition, A2_B_ AdoR also acts on PLC, raising intracellular Ca^2+^ levels.

In a transcriptomic approach, elevated activity of AC was connected to inhibition of AKT signaling and to activation of PKA (41). PKA is important for host defense mechanisms, as inhibition of Salt induced kinases (SIK) by cAMP-activated PKA results in suppression of the pro-inflammatory cytokines IL-6, IL-12, and TNFα in innate immune cells (42–44). Another mediator of suppressive mechanisms along this PKA pathway is the CREB-regulated transcription co-activator 3 (CRTC3). Phosphorylation of CRTC3 is inhibited by the cAMP-activated PKA, leading to translocation of the non-phosphorylated CRTC3 into the nucleus, where interaction with activated CREB upregulates IL-10 gene transcription (43). Parallel to PKA activation, activity of AKT is downregulated by elevated cAMP levels, preventing translation of proteins by means of mTOR inhibition (45). The later pathway plays an important role as regulator of the antibacterial responses in monocytes, macrophages and primary dendritic cells (46, 47). Finally, AdoR-triggered production of cAMP, which is the major second messenger in Tregs after A2-type AdoR engagement does not only convey suppressive downstream signals, it also plays a major role as means of suppression (48). I.e. Tregs suppress other cells by forming gap junctions and transferring cAMP into target cells. Thus, elevated levels of cAMP triggered in Tregs by AdoR engagement may “fuel” the gap junctional suppression (49).

## CD73 in Maintenance of Treg Functions

These suppressive actions of extracellular Ado are operative in almost all A2-type AdoR expressing cells, including Tregs. In particular the conjointly expression of CD39 and CD73 by Tregs enables them to create a self-inhibiting cellular environment, maintaining hyperresponsiveness and stabilizing their own population. For instance, engagement of A2-type AdoR induced expression of Foxp3 and LAG3 in conventional CD4^+^ CD25^-^ T cells, suggesting induction of Tregs (50), and enhanced development of Tregs induced by TGF-β (51). But not only A2_A_ AdoRs are important, as A2_B_R AdoR agonists have been demonstrated to play a role in generation of Tregs, whereas A2_B_ AdoR-deficiency prevented Treg induction (52).

Also direct expansion of Tregs by A2_A_ AdoR-dependent mechanisms has been observed to be important in inflammatory disorders, such as graft-versus-host disease and experimental autoimmune uveitis (51, 53). In *in vitro* cocultures of responder T cells and Tregs the addition of A2-type agonists strongly inhibited activation of cytotoxic effector T cells as expected, but of interest, at the same time significantly enhanced numbers of CD4^+^ Foxp3^+^ Tregs were induced. These cells were highly suppressive and expressed more CTLA-4 molecules (53). Along the same lines, treatment of Tregs with A2-type AdoR agonists before transferring them into an ischemia model enhanced their efficacy *in vivo*, and vice versa A2_A_ AdoR-deficient Tregs were less able to prevent inflammatory damage (51, 54, 55). Even the expression of the Ado-producing enzymes CD39 and CD73 was upregulated by AdoR engagement *via* an E2F-1 and CREB induced pathway (56). Thus, CD73 derived Ado is not only a suppressive means acting on adjacent cells, it also promotes suppressive activity of existing Tregs and generation of “induced” Tregs, and it sustains its own production in a feedback loop by upregulation of CD73 in Tregs.

## Other Than “Ado-Producing” Functions of CD73

### T Cell Signaling

In addition to its enzymatic function, CD73 has been proposed to act as a co-stimulatory molecule for T cells. In conjunction with sub-mitogenic doses of anti-CD3 Abs or PMA, immobilized anti-CD73 antibodies induce proliferation of human peripheral blood T cell, CD25 expression and IL-2 production (57). This qualifies CD73 as a member of stimulatory molecules that transduce activation signals in T lymphocytes (58). Other researchers found that naive CD45RA^hi^ CD45RO^lo^ CD8^+^ T cells that express CD73 have low responsiveness to immobilized anti-CD3, which can be overcome by cross-linking CD3 and CD73 simultaneously (59). The CD73/TCR-signaling complex plays a key role in CD73-mediated activation. This is evident from investigations showing that CD73 transfected mutants of the human Jurkat cell line, which are defective in TCR signaling due to faulty p56^JCK^ or CD45 expression, did not secrete IL-2 after engagement of CD73. In contrast, wild type Jurkat cells transfected with CD73 were activated in response to soluble CD73 mAb plus PMA (60). As for the signal transduction of surface bound CD73, the GPI anchor is not absolutely required, considering that the secreted levels of IL-2 by CD73^+^ Jurkat clones in response to stimulation with CD73 mAb plus PMA were well comparable to that obtained from clones expressing GPI-anchored CD73 (60, 61). As a natural ligand for CD73 has not yet been discovered *in vivo*, all experimental procedures rely on antibody mediated engagement of CD73, which makes it difficult to decipher the role of CD73-mediated activation of T cells *in vivo*. However, Tregs are naturally hyporesponsive, i.e. they do not proliferate, therefore it is not supposed that CD73 conveys mitogenic stimuli in Tregs at all (61).

### Adhesion

CD73 expressed by endothelial cells has a function in mediating lymphocyte adhesion to the endothelium, since CD73-specific monoclonal Ab can inhibit the binding of lymphocytes to endothelial cells (62). Adherent lymphocytes in turn lead to CD73 inhibition on endothelial cells and thus impair the vascular barrier function and facilitate the subsequent steps of transmigration into the tissue (63). Moreover, engagement of CD73 on the surface of lymphocytes indirectly impacts adhesion, as anti-CD73 Ab treatment induces clustering of leukocyte integrin LFA-1 and thereby enhances the adhesion of lymphocytes to cultured endothelial cells (64). Additionally, the anti-CD73 Ab interfered with the binding of germinal center B cells to isolated follicular dendritic cells *in vitro (* 65).

In particular in tumors CD73 seems to be important for migration and dissemination, as the CD73 inhibitor APCP significantly decreases cell adhesion and migration in different breast cancer and hepatocellular carcinoma models *in vitro*, while Ado reversed the effects of APCP (66, 67). However, it is not clear whether these effects are directly mediated by CD73 interactions with cellular ligands or whether CD73-derived Ado is mediating these effects. Nevertheless, although these experiments were performed with T cells or tumor cells in general (not Tregs in particular), it is still conceivable that high and constitutive expression of CD73 by Tregs may act as “homing molecule” for peripheral tissues and CD73^+^ tumors may utilize this mechanism to recruit Tregs.

## Expression of CD73 by T Cell Subsets

Beyond Tregs, almost all T-cell subsets also express CD73, however, to varying degrees. In humans, CD73 is expressed on all CD4^+^ and CD8^+^ T cell subsets, but with higher expression in naïve T cells compared with memory T cells (59, 68). Anergic CD4^+^ T cells that persistently recognize peripheral self-peptide:MHCII complexes and are restrained in their activity through the suppressive actions of Treg cells, share the expression of CD73 with “bona fide” Tregs. This phenotypic trait of CD73 expression enables them to support Tregs to suppress dangerous immune responses to tissue-restricted self-antigens (69). Moreover, uncommitted T helper primed precursor (Thpp) cells were identified in C57BL/6 mice as being CD73^+^ Ly6A/E^-^ CD4^+^ T cells. These cells are able to differentiate into Th1- or Th2- like populations (70), however, a suppressive function is still elusive. Gene expression and phenotypic analysis confirmed that CD73 was enriched in T follicular helper (Tfh) cells, but the utility of CD73 as markers for human Tfh cells has not been fully understood (71).

*In vitro* Th17 cells generated with IL-6 and TGF-β were found to express both CD39 and CD73, leading to Ado formation and subsequent suppression of CD4^+^ and CD8^+^ T cells by limiting IFN-γ and granzyme B production (72). Therefore, acquisition of CD73 expression by otherwise rather proinflammatory T cells, i.e. Th17 cells, can provide them regulatory means. But not only CD4^+^ T cells can acquire a suppressive phenotype by expression of CD73. In humans, naive CD8^+^ T cells express higher levels of CD73 than CD8^+^ memory T cells (59). In line with this, activated CD8^+^ T cells from human peripheral blood cells were shown to lose the membrane expression of CD73 by CD73-containing extracellular vesicles, which have AMPase activity and produced adenosine mediating immune suppression (73). That makes CD73 distribution in tissues independent from Tregs.

Also in mice CD73 is expressed on naïve and memory CD8^+^ T cells and downregulated during terminal effector differentiation (74). CD73^+^ memory T cells are prone to differentiate into cells expressing a tissue-resident memory phenotype since CD73 expression was more frequent by CD69^+^ CXCR6^+^ cells than by CD69^−^ CXCR6^−^ cells (68). Similar to Th17 cells, *in vitro* generated CD8^+^ IL-17-producing T cells (Tc17 cells) were reported to present high levels of CD73 and produce Ado. However, a direct suppressive activity on the proliferation of CD4^+^ T cells was not recorded, but it is involved in maintaining the stem-cell-like properties on Tc17 cells (75).

Finally, even natural killer T (NKT) cells, which are recognizing lipid antigens presented by non-classical class I MHC molecules, express CD39 and CD73 (76). This allows these cells to generate Ado, thus having immunosuppressive properties during tumor and infections (76–78). All of the studies point to a widespread expression of CD73 on T cells, establishing CD73 expression as a major hallmark of a suppressive phenotype of T cell.

## Exosomes Derived From Tumors and Tregs Contain CD73

**Figure 2:**
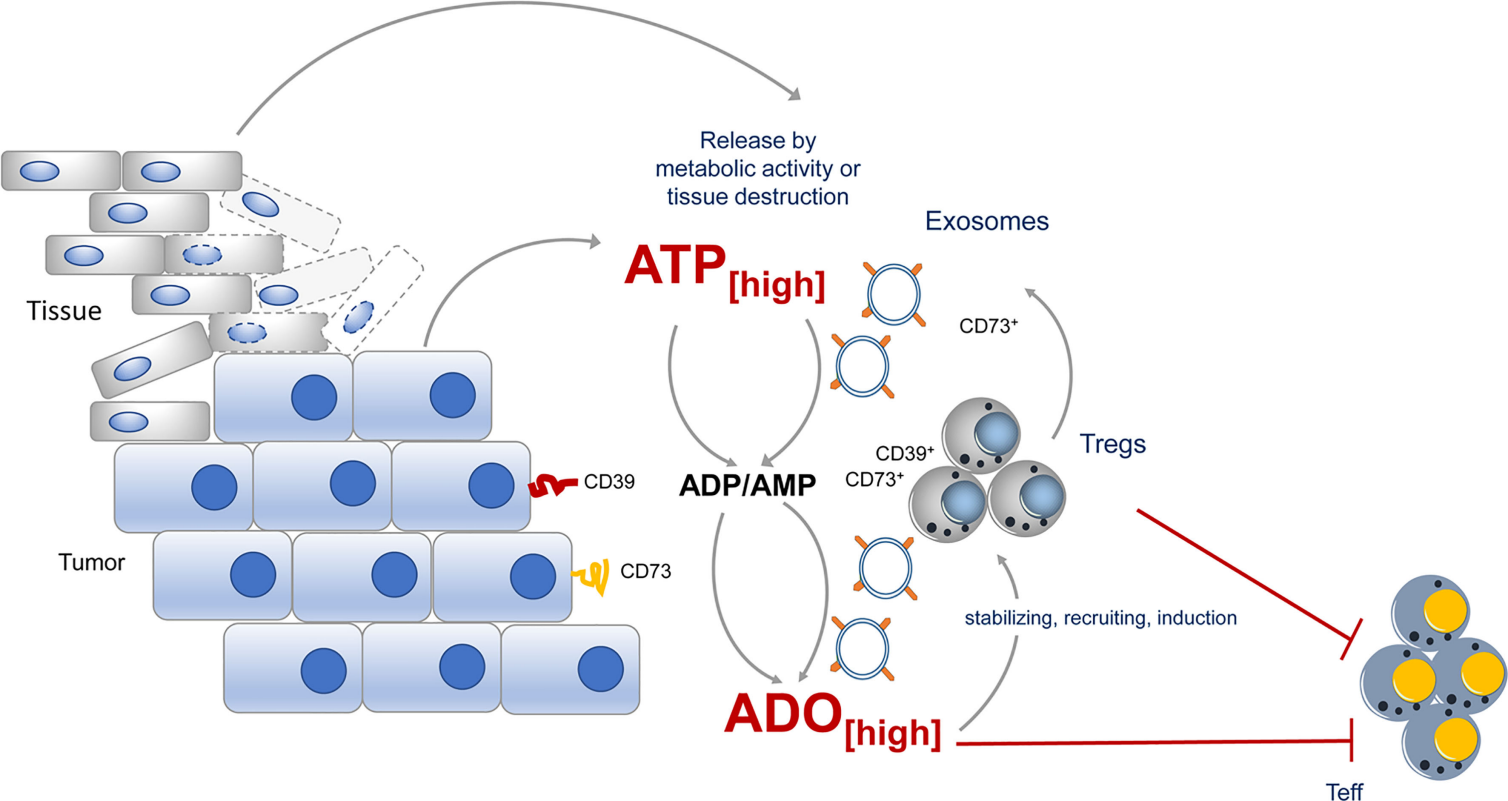
CD73 expression during tumor growth contributes to immune suppression. During tumor growth ATP is either actively release by metabolic active tumors or by dying tissue cells. ATP will quickly be degraded into immunosuppressive adenosine by CD73 expressed by the tumor itself, by incoming Tregs or by exosomes, derived from the tumor or tumor infiltrating lymphocytes. Adenosine maintains the suppressive activity of Tregs in the tumor vicinity and altogether an immunosuppressive environment is favored.

Exosomes are formed from late endosomes, by invagination of multivesicular body membranes, resulting in the formation of intraluminal vesicles (79). During this process, proteins and cytosolic components are taken up into the lumen of the vesicles and subsequently the vesicles are released by fusion with the cell membrane into the pericellular space, becoming “exosomes” (80, 81). Almost all cells produce exosomes for varying purposes. Typically, exosomes are highly enriched in a collection of proteins, lipids, and inhibitory nucleic acids (82), however, expression of tetraspanins, such as CD9, CD63, CD81, CD82 is prototypic for all kinds of exosome vesicles (EVs). Otherwise is the content dependent on the EV-releasing cell type. Different types of cancer cells have captured the mechanism of EV release for immune suppressive actions and CD73 has been identified quite frequently in these “suppressive” EVs (**Figure 2**).

EVs derived from different cancer cell lines express CD73 and catalyze AMP to ado (83). Moreover, B cell-derived CD39^+^ CD73^+^ EVs are elevated in the serum of colon cancer patients and hydrolyze tumor-derived ATP to ado, impairing anti-tumor responses of CD8^+^ T cells (84). Comparably high levels of suppressive activity were also observed in exosomes from pleural fluid of pleural malignant mesothelioma patients. These exosomes trigger a cAMP response in A2_A_ AdoR^+^, but not in A2_A_ AdoR^-^ cells. Significantly elevated cAMP was also induced in Jurkat cells by adding exosomes with ATP but not by adding exosomes or ATP alone (83), indicating that EVs contain the necessary Ado degradation machinery to exert suppression. But not only tumors, also Tregs were observed to release inhibitory EVs. These EVs contain several immunomodulatory molecules, including Treg-specific molecules, such as CD25 and CTLA-4, and of note CD73 (85, 86). As a consequence, proliferation of CD4^+^ T cells and production of IL-2 and IFN-γ was significantly inhibited when Treg-derived exosomes were added to T cell cultures. For these effects CD73 incorporated in EVs was instrumental (85), as exosomes derived from CD73-negative cells failed to convey suppression. Thus, by placing CD73 and other parts of the Ado-generating machinery from the cells surface into EVs, Tregs have developed a method to reach out for suppression and to “export” Ado beyond their immediate cell vicinity (85).

## The Role of CD73 in Tumors and Tumor Infiltrating Tregs

As Tregs are highly positive for CD73, have immunosuppressive activity and are often recruited by tumors to impair anti-tumor immunity, CD73 and its contribution to tumor growth have been investigated thoroughly (**Figure 2**). The expression of CD73 has been found to be upregulated in several types of cancers, including ovarian carcinoma, melanoma, prostate cancer, breast cancer, colon cancer, head and neck cancer, leukemia and glioblastoma (87). Several studies reported that CD73 expression in cancer is associated with a poor prognosis, an increased risk of metastasis and resistance to chemotherapy (88 – 90). For the creation and immune suppressive environment of the tumor, it doesn’t matter whether CD73 is expressed directly by the tumor or by tumor infiltrating lymphocytes; and indeed, as most of the time the tumor is infiltrated by Tregs which express CD73, this Treg-derived “CD73” substantially contributes to elevated levels of immunosuppressive Ado in tumor tissues.

Tregs are CD73^+^ per se, but even further upregulation has been observed in tumors. This may be due to the hypoxic situation in tumors. For long it has been speculated that Tregs might be controlled by tissue oxygen tension. An initial correlation to eAdo is given by the presence of consensus sequences of hypoxia-responsive element (HIF-1α) and cAMP-responsive element in the promoter region of “anti-inflammatory” response genes (91). That could imply that hypoxia and Ado would be responsible for the regulation of CD73 and further immunosuppressive activity of Tregs. Although not definitely decided yet, it is clear that HIF-1α induce Foxp3 in T cells and increase Treg abundance. Notably, HIF-1α-dependent pathways induce upregulation of CD73 in epithelial barriers during hypoxia (92). Thus, the hypoxic tumor environment may provide “the soil” for incoming Tregs to upregulate CD73 and to create an eAdo-dependent immune-suppressive environment.

## CD73 as Immune Checkpoint: Blockage of CD73 as Anti-Tumor Therapy

Due to the strong correlation of CD73 expression with a bad tumor prognosis, CD73 has become a novel immune checkpoint and a target for immunologic intervention. Immune checkpoint inhibitors comprise monoclonal antibodies or small molecule compounds that can target and block inhibitory receptors, and recently anti-CD73 Abs are tested in several (preclinical-) trials (**Table 1**). IPH5301 antibodies were designed to target human membrane bound or soluble forms of CD73, thereby efficiently blocking the degradation of AMP into Ado. Thus, immune cells that are normally targets for Ado-mediated suppression, such as T cells, dendritic cells and macrophages, remained activated and were able to dampen tumor growth in different clinical models (93).

**Table 1:** Current clinical trials targeting CD73*.

Phase	Tumor type	Treatment	Trial #
I	Solid tumors	CD73 inhibitor together with PD-1, A2_A_AdoR antagonist	NCT03549000
I	Solid tumors	CD73 inhibitor together with PD-L1	NCT03835949
I	Solid tumors	CD73 inhibitor together with PD-L1	NCT04148937
I/I	Solid tumors NHL	CD73 inhibitor together with PD-L1, A2_A_AdoR antagonist	NCT03454451
I	Bladder cancer	CD73 inhibitor together with PD-L1	NCT03773666
II	NSCLC	CD73 inhibitor together with PD-L1	NCT03334617
II	Ovarian cancer	CD73 inhibitor together with PD-L1	NCT03267589
I/II	Solid tumors	CD73 inhibitor together with PD-1	NCT02754141
I/II	Pancreatic cancer	CD73 inhibitor together with gemcitabine, paclitaxel, PD-L1, FOLFOX	NCT03611556
I/II	TNBC	CD73 inhibitor together with PD-L1, paclitaxel	NCT03742102

*Antibodies and/or small inhibitors of CD73 functions.

Similar to other already used checkpoint inhibitors, which are applied in combinations, several trials started combinatorial therapies with anti-CD73 Abs (**Table 1**). Here, together with anti-CTLA-4 and anti-PD-1 Abs, anti-CD73 Abs showed significant efficacy in stimulating tumor immunity in several tumor models including colon, prostate, subcutaneous tumors, and breast cancer (94). Interestingly, anti-CD73 Abs preferentially synergized with anti-PD-1 Abs, as activation of A2_A_ AdoR, presumably triggered by CD73-derived Ado, enhances PD-1 expression. Thus, blocking CD73 can interrupt this reciprocal upregulation of Ado and PD-1. However, CTLA-4 expression, on tumor-specific CD8^+^ T cells and CD4^+^ Foxp3^+^ Tregs was not affected by anti-CD73 treatment in this tumor (94). In contrast, anti-CD73 Abs significantly downregulated the expression of PD-1 and CTLA-4 by CD4^+^ and CD8^+^ T cells in a murine transgenic head and neck squamous cell carcinoma (HNSCC) model, resulting in substantial reduction of tumor growth (95). This indicates that different tumor entities may react differently to anti-CD73 treatment.

Yet another already established check point inhibitor, i.e. anti-4-1BB Abs, can profit from combination with anti-CD73 Abs. As anti-4-1BB (CD137) treatment preferentially drives CD73^−^ effector T cell responses for tumor inhibition, combination with neutralizing anti-CD73 Abs induced more profound tumor regression and led to a great increase in survival as compared to anti-4-1BB therapy alone. Likewise, in B16-SIY tumors, combination therapy resulted in an increase in the percentage of tumor-infiltrating CD4^+^ T cells and CD8^+^ IFN-γ^+^ double positive T cells, whereas a significant decrease in the percentage of tumor infiltrating Foxp3^+^ Tregs was observed in anti-4-1BB/anti-CD73 Abs-treated mice (96).

However, anti-CD73 Abs in therapies have to be analyzed individually, as for instance the TGF-β-rich tumor milieu confers resistance to anti-4-1BB therapy by sustaining CD73 expression, primarily on infiltrating CD8^+^ T cells across several tumor models, and making them rather immunosuppressive. Therefore, even additional blockade of TGF-β may be beneficial for downregulation of CD73 expression on infiltrating T cells, and together with anti-CD73, Abs resistant tumors are rendered sensitive to agonistic anti-4-1BB therapy (96).

Beyond the mere blockade of Ado production by anti-CD73 therapy, murine tumor models also confirmed beneficial effects on the ability of tumors to metastasize and to induce tumor cell migration. For instance, in a mouse model of human breast cancer, anti-CD73 Abs changed cellular adhesion and migration patterns, by inducing clustering and internalization of surface-expressed CD73 (97). These effects were independent of hematopoetic cell involvement (97–101) and are reminiscent of “adhesion data” discussed in a previous section. Therefore, these studies point to a non-catalytic role of CD73 expressed on tumor cells and/or endothelial cells, and anti-CD73 Abs can positively influence tumor cell adhesion, extravasation and metastasis to support anti-tumor immunity.

## Conclusion

CD73, the ecto-5-nucleotidase, is constitutively expressed on Tregs which can convert AMP to immunosuppressive Ado. A worse tumor prognosis has been found to be strongly associated with Ado production by CD73 in cancer. Thus, targeting CD73 becomes a novel method to improve anti-tumor immunity by combining to anti-CTLA-4, anti-PD-1 or anti-CD137 Abs.

## Author Contributions

MD and LC wrote the first draft of the paper and prepared figures. SR and KM corrected the draft and the figures. AE drafted figures and tables and corrected the manuscript. All authors contributed to the article and approved the submitted version.

## Funding

This work was supported by the Deutsche Forschungsgemeinschaft TR156 B03 and C04. MD and LC are supported by a scholarship CSC 201806090291 and 202006320072, respectively. Publication was supported by the Deutsche Forschungsgemeinschaft within the funding programme “Open Access Publikationskosten” as well as by Heidelberg University.

## Conflict of Interest

The authors declare that the research was conducted in the absence of any commercial or financial relationships that could be construed as a potential conflict of interest.

## Publisher’s Note

All claims expressed in this article are solely those of the authors and do not necessarily represent those of their affiliated organizations, or those of the publisher, the editors and the reviewers. Any product that may be evaluated in this article, or claim that may be made by its manufacturer, is not guaranteed or endorsed by the publisher.
